# Western Corn Rootworm, Plant and Microbe Interactions: A Review and Prospects for New Management Tools

**DOI:** 10.3390/insects12020171

**Published:** 2021-02-17

**Authors:** Kyle J. Paddock, Christelle A. M. Robert, Matthias Erb, Bruce E. Hibbard

**Affiliations:** 1 Division of Plant Sciences, University of Missouri, Columbia, MO 65211, USA; paddockk@mail.missouri.edu; 2 Institute of Plant Sciences, University of Bern, 3013 Bern, Switzerland; christelle.robert@ips.unibe.ch (C.A.M.R.); matthias.erb@ips.unibe.ch (M.E.); 3 Oeschger Centre for Climate Change Research, University of Bern, 3013 Bern, Switzerland; 4 Plant Genetics Research Unit, United States Department of Agriculture, Agricultural Research Service, Columbia, MO 65211, USA

**Keywords:** Western corn rootworm, belowground herbivory, pest management strategies, push-pull, plant defenses, biological control, soil health

## Abstract

**Simple Summary:**

Over 90 million acres of US cropland are planted with corn, *Zea mays*, annually. The western corn rootworm, *Diabrotica virgifera virgifera*, causes significant economic damage by feeding on corn roots and the insect has populations that have adapted to nearly all management techniques in some regions. Additional tools are needed. Significant research on the basic biology of this pest has added new possibilities. Here, we summarize research that we believe has potential for future management of this major pest.

**Abstract:**

The western corn rootworm, *Diabrotica virgifera virgifera* LeConte, is resistant to four separate classes of traditional insecticides, all *Bacillius thuringiensis* (Bt) toxins currently registered for commercial use, crop rotation, innate plant resistance factors, and even double-stranded RNA (dsRNA) targeting essential genes via environmental RNA interference (RNAi), which has not been sold commercially to date. Clearly, additional tools are needed as management options. In this review, we discuss the state-of-the-art knowledge about biotic factors influencing herbivore success, including host location and recognition, plant defensive traits, plant-microbe interactions, and herbivore-pathogens/predator interactions. We then translate this knowledge into potential new management tools and improved biological control.

## 1. Introduction

Management of the western corn rootworm (*Diabrotica virgifera virgifera* LeConte) in maize cropping systems has a long, complex history. After its discovery as a pest of corn in 1909 [1], western corn rootworm (WCR) populations rapidly expanded eastward as corn was planted, reaching New England and, more recently, through multiple establishments and subsequent spread, European regions [2,3,4]. Annual costs of damage due to yield loss and management practices are estimated to be over $2 billion in the United States [5]. A diversity of management options exists [6], but management has been complicated by the continual adaptation of WCR to control tactics. WCR have developed resistance to four separate classes of traditional insecticides, all Bt toxins currently registered for commercial use, crop rotation, and even to dsRNA and innate plant resistance traits [7,8,9,10,11,12,13,14,15,16,17]. In some regions of Europe, management options are even more limited as use of transgenic maize producing Bt targeting rootworms are restricted, and limitations have been placed on neonicotinoids [18].

New tools for sustainable and economical management of this elusive pest are crucially needed. In this review, we highlight the status and potential of several prospective tools based on recent advances in the understanding of the biology and chemical ecology of the pest. These tools include push-pull strategies, plant defenses and nutrition, beneficial plant-microbial partners, and microbial control agents.

## 2. Disrupting WCR Establishment

Considerable efforts investigating the chemical ecology of maize-rootworm interactions have illuminated complex mechanisms, including physical and chemical processes, involved in host plant attraction, recognition, and in feeding stimulation. WCR larvae hatch in spring from eggs laid in the previous year. The period between eclosion and host plant establishment is critical for WCR. It is estimated 95% of hatching larvae die before establishment, but the factors responsible for high mortality remain unknown [19,20,21]. Physical and chemical factors have been demonstrated to affect first-instar larval movement and host plant establishment. Larval movement is limited with increasing soil bulk density [22,23] with first instar larvae traveling farther in finer textured soils compared to more coarse textured soils [24]. Increased egg distance to maize roots can limit root damage and adult emergence [25]. Questions still remain as to why most viable eggs fail to produce established larvae. For example, it is unknown the extent to which first-instar larvae can burrow through soil and instead rely on pre-existing soil pores and air channels [26]. If this major mortality factor was better understood, it might be possible to manipulate it for management.

It may also be possible to utilize knowledge of the factors influencing host plant establishment for management in the future. WCR larvae orient towards maize roots following CO_2_ gradients and can detect concentrations as low as 2 mmol/mol [27,28,29]. In choice tests, significantly more neonate WCR larvae were attracted to synthetic CO_2_ with a concentration of 11.2 mmol/mol than to growing maize with a CO_2_ concentration of 1.36 mmol/mol [29]. Encapsulated CO_2_ sources were tested as a means to confuse western corn rootworm larvae. The treatment resulted in significantly less damage than untreated controls and resulted in damage similar to a soil insecticide control [30]. CO_2_ has also been evaluated with insecticides in an attract-and-kill approach [31]. However, neither approach has been adopted by industry. Other volatiles, including *(E)*-β-caryophyllene and ethylene, can also be detected and used by the WCR to locate suitable hosts [32]. For instance, *(E)*-β-caryophyllene can be used as a cue to orient towards roots attacked by conspecific larvae and to aggregate in a density-dependent manner [33]. *(E)*-β-caryophyllene does not attract neonates [34], so its usefulness as a volatile to confuse WCR larvae in the field remains unclear.

Once maize roots are located, larval feeding is triggered by host recognition cues. Strnad and Dunn [35] were the first to document the existence of host recognition factors. One such recognition factor was isolated [36] and identified by Bernklau et al. [37] as monogalactosyldiacylglycerol (MGDG). Bernklau et al. [37] discovered that the proportion of larvae exhibiting the tight-turning behavior elicited by MGDG was higher for larvae exposed to MGDG-saturated discs previously fed upon by WCR larvae. The authors concluded that WCR larvae were responding to byproducts of MGDG breakdown in addition to MGDG itself, which generates questions in regard to salivary enzymatic functions and plant-insect interactions involving WCR. Previously, Bernklau and Bjostad [38] isolated and identified a blend of glucose (30 mg/mL), fructose (4 mg/mL) and sucrose (4 mg/mL) plus linoleic or oleic acid (0.3 mg/mL) as feeding stimulants for WCR larvae. Subsequent investigations revealed sucrose to be the preferred sugar of WCR larvae [39]. The addition of free fatty acids to feeding blends significantly increased staying behavior of larvae, but high concentrations were toxic [40]. In addition to primary metabolites, complexes between micronutrients and maize secondary metabolites shape the foraging behavior of the WCR within a root system [7,41]. Specifically, complexes between soil iron (Fe) and the exuded 7-O-methylated, N-hydroxylated benzoxazinoid (DIMBOA) elicited WCR feeding preferences [41]. Interestingly, the application of the Fe(III)(DIMBOA)_3_ complex on rice or barley, two non-host plant species for the WCR, was sufficient to trigger WCR feeding [41]. Experiments with benzoxazinoid-deficient plants and WCR larvae with impaired capacities to detect sugars confirmed the importance of the individual and combined cues for WCR foraging, but also revealed considerable WCR robustness to disruption of individual cues [42]. This may complicate attempts to use single cues for foraging disruption. Plant roots also produce compounds that repel foraging WCR larvae. Bernklau et al. [43] identified that small amounts (1 ug) of methyl anthranilate could prevent WCR larvae from approaching CO_2_ sources and maize roots. Although the identification of compounds involved in feeding stimulation and staying behavior are useful, a suite of compounds is likely at play, as responses to crude maize extracts are generally stronger.

This basic understanding of attraction, recognition, and feeding stimulation could be utilized to improve existing management strategies or aid the development of alternative control tactics. Strategies such as (i) fine-tuning the production of cues involved in WCR attraction, establishment, and feeding, (ii) combining attractants with pesticides, or (iii) using attractant and repellent chemicals in push-pull programs should be considered. Manipulating the production of attractants, recognition factors, or feeding stimulants remains a very delicate avenue for pest management. One should carefully consider the impact of any shifts in primary or secondary plant metabolites on the plant and their interactions with the environment. For instance, because maize plants use Fe(III)(DIMBOA)_3_ complex for iron uptake and benzoxazinoids for protection against generalist herbivores, disrupting the benzoxazinoid pathway for WCR management could potentially have significant consequences on plant growth and yield, as well as on herbivore outbreaks [44]. The application of attractant volatiles can disrupt host location and, in turn, establishment and damage by the WCR. CO_2_-generating materials are strong enough to disrupt the host-location ability of WCR larvae and significantly reduce damage under laboratory and field conditions [30]. Combining attractant cues with pesticides has been reported to be extremely effective in a laboratory setting. For example, Bernklau et al. [45] increased insecticidal activity of thiamethoxam by 10,000-fold when added to a feeding stimulant blend. The addition of 6-methoxy-2-benzoxazolinone (MBOA) to insecticides also improved field activity [46]. Adding host recognition cues, feeding stimulants, CO_2_, or other attractants to insecticides could increase efficacy and perhaps even provide a “pull” factor for a push-pull management strategy [47]. Bernklau et al. [48] documented that methyl anthranilate acts as a repellent for foraging WCR larvae in soil. Methyl anthranilate-saturated carriers could be placed in-row with maize seedlings and function as a “push” factor away from roots. Similarly, susceptible corn with repellent seed treatments could be used in conjunction with high-dose transgenic corn treated with attractants and/or feeding stimulants. Push-pull strategies have been used successfully in pest management with other crop systems [47].

## 3. Selecting for Maize Lines with Effective Defenses against WCR

Selecting for plant natural defenses to insects has been a successful management strategy for many pests, with hundreds of insect-resistant crop cultivars grown around the world [49,50]. Plant defenses include resistance and tolerance traits. Resistance traits allow plants to reduce herbivore damage [51,52] and are further categorized into antibiosis and antixenosis. Antibiosis refers to a reduction in growth and/or reproduction of the insect due to feeding on a resistant plant, whereas antixenosis limits damage to the host plant by decreasing the attractiveness of the host as food or shelter. Resistance traits might involve structural changes (root architecture, lignin content, trichomes) or production of allelochemicals (tannins, alkaloids, glucosinolates) [49,53,54,55]. Tolerance traits allow the plant to maintain productivity in spite of sustaining damage [56]. Tolerance to root herbivores, for instance, includes changes in photosynthesis, resource reallocation, and delayed compensatory growth [57,58,59,60].

Public breeding efforts to develop or select plant defenses to WCR have been intermittent for the past 85 years (Table 1) [61,62,63,64,65,66,67,68,69,70,71,72,73,74,75,76,77,78,79,80,81,82,83,84,85,86,87,88,89,90,91,92,93,94,95,96,97,98,99,100,101,102,103,104,105,106,107,108,109,110,111,112,113,114]. Initial breeding programs began in response to observations that different maize strains varied in their response to WCR pressure [61,62]. In recent breeding programs, selection for resistant and tolerant strains of maize has been based on several criteria. These criteria include plant lodging, vertical pull resistance, and yield, which serve as indirect measures of WCR damage. Root damage ratings and WCR survival provide an estimate of antibiosis and/or antixenosis capacity. Root size and root regrowth, although influenced by environmental factors, provide an estimate of tolerance to WCR damage. Early resistant hybrids (1980s) had larger roots and experienced lower levels of lodging upon WCR feeding [80]. More recently, several germplasm lines with mechanisms of resistance beyond tolerance have been identified [75,85,92,94,99,100,104,113]. Hibbard et al. [94] released CRW3(S1)C6 that had damage ratings not significantly different than a Cry3Bb1 hybrid when crossed to an elite inbred line. El Khishen et al. [99] and Bernklau et al. [100] clearly documented that the commercial maize hybrids SUM2162 and SUM2068 had relatively strong antibiosis resistance. Unfortunately, these hybrids did not compete with elite hybrids for yield when rootworm pressure was lacking, and at this time, there are no commercially available hybrids providing natural and effective host plant resistance or tolerance to WCR.

Genomic work in maize has revealed a bounty of natural diversity across germplasm lines [115]. Screening of landraces, populations, and inbreds by insect-resistance breeding programs has revealed that genetic bins containing insect resistance quantitative trait loci (QTLs) are widespread, likely meaning there is great complexity and diversity in maize responses to herbivores [116]. However, because much of the work of insect-resistance breeding programs has focused on stem and leaf feeding traits, confirmed resistance against leaf herbivores is more prevalent than resistance to root feeding insects. This does not necessarily exclude these QTLs from conferring resistance to root-feeding insects. Recent work from Bohn et al. [107] revealed that QTLs associated with differences in root damage by WCR overlapped with QTLs involved with insect resistance previously identified by Meihls et al. [116]. Many of these QTLs (chromosome 1, 3, 6–10) contained gene/genes predicted to code for proteins involved in L-ascorbate and (*E*)-β-caryophyllene biosynthesis, in addition to the detoxification of reactive oxygen species. Investigations by Brkić et al. [114] found chromosome 1 and 6 contain several QTLs for maize resistance to WCR. Studies investigating the QTL regions previously described may provide a knowledge base for breeding programs aimed at increasing maize native resistance to WCR. Specifically, the QTLs correlated with resistance to WCR were located in the same genomic bins as two previously described insect resistance genes, *aoc1* (bin 1.04) and *mir1* (bin 6.02). *mir1* encodes an insecticidal protease, Maize Insect Resistance 1- Cysteine Protease (MIR1-CP), that can disrupt the peritrophic matrix of caterpillars and even act synergistically with Bt toxins [117,118]. Separate investigations have revealed *mir1* expression increases upon WCR feeding in the inbred Mp708 [119]. In addition, transcript levels of several defense genes (*tps23*, *fpps3*, *rip2*, *mpi*) significantly increased in conjunction with jasmonic acid (JA) levels, potentially contributing to reduced larval recovery and reduced root damage [119]. Unfortunately, Castano-Duque et al. [119] did not utilize resistant and susceptible maize controls, so it is unclear how well this resistance would translate into a field setting. Other data suggest Mp708 is highly susceptible to natural rootworm feeding in the field (BEH, unpublished data). Given the high degree of WCR host adaptation, we estimate that the efficacy of generalized defense traits present across most commercial maize lines have limited potential to serve as WCR resistance factors.

As mentioned above, there are no publicly available hybrids conferring natural resistance or tolerance to WCR damage. The lack of correlation between inbred performance and hybrid performance [98] likely has contributed to this. Genotype-by-environment interaction (GEI) is high for natural rootworm resistance, resulting in low heritability of traits [107]. Likely contributing to GEI variability are differences in methodology. Infestation levels (natural variability vs. artificial) can generate high amounts of variation between environments and drown out trait effects. The paucity of effective natural defenses against WCR in commercial hybrids could also be a function of private breeding programs largely controlling for the pest via crop rotation or soil insecticides in their yield trials. This effectively removed the selection pressure and potentially decoupled yield and WCR tolerance/resistance traits. In contrast, seed industry yield trials rarely control for herbivores such as the European corn borer, *Ostrinia nubilalis* (Hübner), and therefore indirectly increase tolerance to this pest over time (James Bing, Corteva, personal communication). Despite the lack of current resistant and/or tolerant hybrids available to growers, native resistance traits from exotic sources likely do have potential to improve WCR management. Full genome sequencing and improved breeding methods coupled with modern gene editing technologies examining direct effects of specific gene/genes involved with defensive traits could potentially increase the speed and efficiency of elite cultivar development with native plant defense [120]. Ultimately though, the success relies on investing time in screening these plants for their capacity to cope with WCR damage.

## 4. Altering Maize Nutritional Value for the WCR

The nutritional dimension of WCR biology is central to management, but research efforts to understand this aspect have been intermittent. Assessing chemical profiles of host and non-host plant species may allow determination of key compounds or compound blends involved in WCR nutrition. Branson and Ortman [121,122] observed larval survival for at least 10 days on grass species. WCR neonates developed to the second instar on 18 of 44 grass species screened, whereas no larvae developed on any of the 27 broadleaf species screened. Clark and Hibbard [123], Oyediran et al. [124], and Wilson and Hibbard [125] further refined the host range of WCR larvae. Moeser and Vidal [126] developed a food conversion index to evaluate alternate hosts and several maize varieties. Selecting for maize lines possessing some key characteristics of non-host plants may limit WCR damage in the field.

Evaluating WCR larval ability to pupate and to emerge as adults when feeding on maize plants of different ages showed promising results. WCR consume root resources near where they initially establish, before moving to larger, more nutritious nodal roots that form on the side of the stalk [7,22]. Not only do later instar larvae prefer younger, nodal roots, but larvae require these younger roots for proper development [127]. Hibbard et al. [127] conducted greenhouse and field trials to determine what root phenology was optimal for the establishment and development of WCR larvae. In the field, plants were infested weekly with WCR eggs starting on the initial planting date and continuing until plants matured to ~V13 [128]. As predicted, plant damage gradually decreased with later infestation dates, because larger root systems can better withstand attack. Interestingly, larval recovery did not differ between infestation dates, but adult emergence did. Significantly fewer adults emerged from later infestation dates, suggesting larvae can establish on late vegetative stage (V13) plants, but nutrition is insufficient to produce adults. Given that WCR larvae perform poorly in the absence of Fe(III)(DIMBOA)_3_ complexes and that DIMBOA is mostly exuded by young node roots of young plants [7,41], it is tempting to speculate that iron, known to be an essential micronutrient for insects [129], and/or DIMBOA are key factors in limiting WCR development to adulthood. Results from alternative host plant species also point towards nutritional inadequacies of mature plants [130]. Further understanding of WCR nutritional requirements to successfully achieve pupation may allow selection for plants that do not support WCR larval development to adults.

Further efforts to understand the nutrition requirements of WCR larvae have resulted in the development of artificial diets [131]. Current efforts to improve artificial diet further are focused on understanding the metabolomic responses to maize, in addition to good and poor artificial diets (Huynh et al., unpublished). If successful in gaining this understanding, reverse engineering maize varieties with roots of poor nutritional quality may also be possible. Changing such traits is typically accompanied by large pleiotropic effects, as herbivore nutrients also serve essential roles in plants. Thus, such approaches should try to disrupt WCR nutrition without impairing plant vigor; strategies to reach this aim are currently not in place.

## 5. Plant-Mediated RNA Interference

RNA interference (RNAi) is a biological response to double-stranded RNA (dsRNA) that triggers sequence-specific gene silencing [132]. This conserved machinery is present in many eukaryotes, including insects [133]. Silencing essential genes in insects can reduce herbivore damage and survival [134,135,136,137,138,139].

Baum et al. [140] and Bolognesi et al. [141] characterized the mechanism of action of dsRNA in WCR larvae. Baum et al. [140] identified 125 genes whose silencing led to significant WCR mortality. From these 125 genes, 14 caused mortality in 50% (LC_50_) of WCR at doses lower than 5.2 ng dsRNA/cm^2^ of artificial diet. These genes included putative *V- ATPase* A and D subunits, *ESCRT I Vps28*, *III Vps2*, and *III Snf* orthologs, a β-subunit of a *COPI* coatomer, ribosomal proteins, a proteosome ortholog, *α-actin*, *tubulin*, and an *RNA polymerase II* ortholog [140]. Adult WCR exhibit similar responses to orally ingested dsRNA. Using artificial diet overlaid with dsRNA targeting *V-ATPase* subunit A, Rangasamy and Siegfried [142] successfully knocked down gene expression in adults and achieved significant mortality in 14 days. Knockdown of the gene target *Sec23* resulted in significant mortality in adults after only six days of feeding [143]. Additional gene targets have successfully altered adult gene expression, specifically ones targeting genes involved in reproduction such as the chromatin remodeling gene *brahma* (brm), and the gap gene *hunchback* (hb) [144,145]. Eggs from RNAi targeted adults also experience downregulation of targeted genes, which could provide transgenerational control [144]. RNAi is now routinely used to identify key genes regulating WCR survival and fitness [41,144,146,147].

Transgenic maize plants using RNAi exhibit enhanced protection against WCR larvae when targeting essential genes such as *α-tubulin* gene, *V-ATPase* subunits A and C genes, an intracellular protein trafficking pathway gene *snf7*, a subunit of the coat protein complex II *Sec23*, and a midgut expressed gene *ssj1* [140,141,143,147,148]. In addition, Niu et al. [149] demonstrated the potential for WCR management by silencing genes involved in female fecundity. Using transgenic plants to silence the *Boule* (*Dvbol*) gene in WCR larvae resulted in reduced egg production and egg hatchability in adults [149]. Yet, Khajuria et al. [16] demonstrated the ability of the WCR to adapt to RNAi. WCR selected on *DvSnf7* dsRNA displayed an impaired luminal uptake [16]. Intriguingly, *DvSnf7* dsRNA resistant WCR also displayed cross resistance against three other dsRNA sequences but not to the *Bacillus thuringiensis* Cry3Bb1 protein [16].

Transgenic crops using RNAi may therefore be a promising tool when combined with other strategies for pest management. Bayer Crop Science and Corteva developed new transgenic lines expressing the combination of microbial compounds with RNAi targets [150,151] (see the section below about pathogenic microbials). Large scale application and potential resistance development in the field have yet to be evaluated. Deployment of stacked dsRNA targeting immature and adult life stages might better capture WCR surviving single traits. However, efficacy of transgenic plants expressing dsRNA against adult WCR has not been thoroughly evaluated.

## 6. Enhancing Plant Health-Promoting Microbes

Plant-insect-microbe interactions occurring in the rhizosphere can have dramatic effects across trophic levels, above and below ground, and can shape plant, herbivore, and microbial communities [152]. Beneficial microbial communities can provide plants with increased pest resistance [153]. Plants release a suite of chemicals from roots upon insect damage to which specific microbes respond. Over multiple generations, plants can then refine microbial communities that provide beneficial functions [154]. In the case of maize, benzoxazinoids have been shown to alter the rhizosphere microbiome, providing potential benefits to maize in the form of pest suppression in following generations [155]. Organic practices that promote soil health can also alter plant resistance to aboveground pest pressure [153]. However, there has been little work to investigate how these rhizosphere interactions affect western corn rootworm. Several groups are currently evaluating the potential of root-associated microbes to manage WCR. As a soil-dwelling root herbivore, WCR larvae are likely well adapted to maize rhizosphere microbial communities. Therefore, introducing non-native microbes that are compatible with maize, but not WCR physiology, may be a promising path.

Arbuscular mycorrhizal fungi (AMF) are one of the most integral groups of microorganisms promoting plant health. It is estimated >80% of plant species form symbiotic relationships with AMF [156]. Plants associated with AMF exhibit increased nutrient absorption of P and other micronutrients [157]. AMF colonization can also increase induced jasmonic acid pathways involved in plant defense against herbivores [158]. However, predicting whether AMF colonization negatively or positively affects herbivore performance is complex [159]. A meta-analysis by Koricheva et al. [160] revealed chewing insects experience reduced performance on AMF associated plants while piercing-sucking insects experience an increase in performance. Many mechanisms are likely at play as AMF can reconfigure the plant primary and secondary metabolisms [161]. Jaffuel et al. [162] examined protection ability of a seven-species AMF seed treatment against WCR in the field. AMF treatment had no effect on root damage, WCR fitness, or yield. The extent of AMF association with roots was not measured, which makes predictions about AMF species effects difficult to interpret. Future investigations should consider promoting native AMF abundance and examine species-specific responses of WCR to AMF-colonized maize. Winter cover crops increase soil health by reducing erosion, limiting nutrient loss, and increasing microbial abundance and diversity [163,164]. Different species of cover crop can refine AMF and other microbial communities in distinct ways [165,166,167,168], and the legacy effects of cover crops can increase AMF colonization of cash crop roots [158,167]). Winter cover crops can also increase predator populations and correlate to reductions in root damage and WCR larvae [169]. This broader approach through ecological intensification could be employed by combining management techniques to sustainably manage populations and reduce damage. Work in this field is in its infancy but has potential to expand into new management applications.

## 7. Using Soil Microbials to Disrupt WCR Gut Microbiome

Douglas [170] theorized the exploitation of insect microbiomes could provide new pest management techniques. Studies have shown the western corn rootworm actively selects for microorganisms it harbors [171,172]. Larvae reared in two different soils harbor similar bacterial communities even though the soil samples vary widely in community composition [172]. The WCR bacterial community commonly consists of species of *Serratia*, *Pseudomonas*, *Klebsiella*, *Acinetobactor*, *Streptomyces*, and *Tsukamurella*, with other species appearing in high abundance but sporadically [171,172,173,174]. Studies have focused on surveying the bacterial community of WCR but have done little to try to characterize functionality of that community. Robert et al. [175] evaluated fitness of multigenerational antibiotic-treated WCR and found no significant difference in weight gain or survival on conventional corn. Antibiotics were given to adults, and only the presence of *Wolbachia* was analyzed using PCR, so it is unclear what other bacteria remained after antibiotic treatment, or what bacteria were acquired from the soil during larval feeding. A majority of WCR populations also carry a high proportion of the maternally transmitted endosymbiont, *Wolbachia* [171,172,176]. *Wolbachia* can play an influential role in insect reproduction by inducing cytoplasmic incompatibility, parthenogenesis, feminization, and male killing [177]. Reproductive isolation caused by cytoplasmic incompatibility can result in speciation events at a much greater speed than traditional genetic elements [178,179]. Two subspecies of *Diabrotica virgifera*, *D. v. virgifera* (WCR) and *D. v. zeae* Krysan and Smith (Mexican corn rootworm), are a result of *Wolbachia*-induced cytoplasmic incompatibility that occurred after the ancestral population reached the area of modern-day Arizona less than 1100 years ago [180,181]. *Wolbachia* has also been shown to influence the composition of the host microbiome [182] and even protect the host from viral infection in populations of *Drosophila melanogaster* [183]. A role outside of reproductive incompatibility has not been found for *Wolbachia* in WCR. *Wolbachia* does appear to modulate plant gene expression, but this does not seem to impact major defenses or WCR resistance [175,184]. Nonetheless, it appears that some of the bacteria that inhabit WCR display functional capacity in overcoming plant defenses. Chu et al. [173] identified alterations in the gut microbial community of rotation-resistant populations of WCR. These shifts in the bacterial community were accompanied by increased cysteine protease activity in the gut that facilitated adult survival on soybean foliage [173]. In another study, bacterial isolates from abdomens of females influenced oviposition preference in choice tests [185]. The number of examples illustrating the role of the herbivore microbiome in overcoming plant defenses in other systems is increasing [186,187,188,189].

Mechanistic studies investigating the role of WCR gut microbiome in WCR ecological success are crucial to develop effective pest management strategies. A possible avenue would be to inoculate the soil with specific microbes that would shift WCR gut microbiome communities and hinder their ability to overcome plant defenses.

## 8. Using Pathogenic Microbials to Reduce WCR Populations

Management of arthropods through the use of microbes has a long history [190,191]. More recently, greater emphasis has been placed on limiting damage to non-target organisms and, in turn, has significantly increased the attractiveness of microbes as biocontrol agents. Classically, there are five main categories recognized under the term microbial control agents (MCAs): bacteria, viruses, fungi, protozoa, and nematodes [192]. Each has a unique mode of action and requires careful application to maximize benefit within the integrated pest management (IPM) framework. As advancements have been made in the microbial control of several pest species, their applications for WCR have mostly focused on transgenic approaches and entomopathogenic nematodes (EPNs).

The entomopathogenic bacteria from the genus *Bacillus* are some of the most widely used microbial biocontrol agents. These bacteria produce crystal toxins (Cry) that cause mortality in the insect by inducing cell lysis in the midgut [193]. Corn rootworm management has largely depended on *Bacillus thuringiensis* (Bt) for several decades through the planting of transgenic corn that express Cry toxins. There are currently four different Bt toxins commercially available as in-plant corn traits. In addition, Bayer Crop Science has developed SmartStax PRO expressing Cry3Bb1, Cry34Ab1/Cry35Ab1 and DvSnf7 (*Diabrotica virgifera* (Dv) + sucrose-non-fermenting (SNF) locus), a novel RNAi-based trait which targets a specific RNA sequence of WCR [150]. This product is approved by the EPA and recently received import approval from China. These germplasm will be widely planted for the first time in 2022. Bayer also recently discovered an additional Bt protein [194] and a protein from *Brevibacillus laterosporus* [195], each with strong activity against western corn rootworm larvae and no cross resistance to current rootworm toxins. Corteva has a new transgenic maize line producing a toxin derived from *Pseudomonas chlororaphis* pyramided with dsRNA available [151], but the feasibility for large scale application has yet to be evaluated. Other toxins displaying activity against WCR have also been documented. These toxins were originally isolated from the entomopathogenic bacteria, *Chromobacterium piscinae*, *Pseudomonas mosselii*, *Alcaligenes faecalis*, *Photorhabdus luminescens* [196,197,198,199], and entomopathogenic fungi from the genus *Pleurotus* [200]. Examination of bacterial species that display toxicity in other Coleopteran species (*Yersinia entomophaga*, *Paenobacillus spp.*, *Serratia entomophila*) are lacking for the western corn rootworm [191,201,202,203].

Entomopathogenic virus research has largely focused on the family Baculoviridae. This diverse viral family has shown promise as an MCA for Lepidopterans but seems to lack efficacy in Coleopterans. As such, Coleopteran viral research has focused on non-Baculoviridae species. Liu et al. [204,205,206] have identified two single-strand RNA viruses and an iflavirus present in WCR, but functional characterization has yet to be elucidated. Some success using virus as an MCA in beetles was demonstrated with the *Oryctes rhinoceros nudivirus* control coconut palm rhinoceros beetle (*Oryctes rhinoceros*) [207]. Fungi have also been under-evaluated as an MCA in corn rootworm. Strains of *Metarhizium anisopliae*, *Beauveria bassiana*, and *Beauveria brongniartii* display toxicity in larvae and adults [208], and *M. anisopliae* can significantly reduce adult emergence in field settings [209]. *B. bassiana* is commonly available as an MCA from several biopesticide companies, but it remains to be seen if treatments can be an economically viable control measure for WCR. Alternatively, both *M. anisopliae* and *B. bassiana* are found in agricultural soils and with proper soil management, could serve as a type of conservation biocontrol [210].

Entomopathogenic nematodes from the families Steinernematidae and Heterorhabditidae display high virulence against WCR [211] and, consequently, have been used to limit damage to maize infested with WCR [162,212,213]. Infective juveniles have a relatively short shelf life, making formulations difficult to use [214]. However, inducing a state of quiescence can prolong the infectiveness of EPN juveniles [215]. Root cap extracts of maize and pea contain potent amounts of quiescence factors that could be utilized to increase shelf life [216]. In addition, releases of nematodes for control rely on annual releases. No published studies have examined the long-term persistence of EPNs in WCR-maize systems, but such evidence exists in other systems [217]. Furthermore, field formulations and WCR specific strains show promising effects in the field and are available commercially [218,219,220].

Understanding the ecology of EPNs and identifying the infochemicals involved in their success will facilitate development of improved IPM strategies [221]. EPNs locate their host using universal, plant- and insect-derived chemical cues [222]. For instance, *H. megidis* can use the herbivore-induced root volatile *(E)*-β-caryophyllene [223] but see [224]. Hiltpold et al. [225] showed that EPNs can be selected for increased responsiveness towards the terpenoid volatile within six generations. The enhanced EPN responsiveness to *(E)*-β-caryophyllene increased EPN success in controlling WCR populations, but only to maize hybrids that emit the volatile. *H. bacteriophora* is strongly attracted to WCR cadaver cues, such as butylated hydroxytoluene [226]. Interestingly, EPN-infected cadavers are not only attractive to EPNs but also to the herbivores themselves [226,227] and trigger a plant defensive response [228,229].

WCR larvae can redirect plant defenses against nematodes [230]. Specifically, WCR larvae accumulate two benzoxazinoid glucosides: 6-methoxy-2-benzoxazolinone N-glucoside (MBOA-Glc) and 2-hydroxy-4,7-dimethoxy-1,4-benzoxazin-3-one O-glucoside (HDMBOA-Glc). MBOA-Glc is exuded by WCR onto its cuticle and is present in large amount in its frass [230]. This insect-specific detoxification benzoxazinone repels the EPN, *H. bacteriophora*. HDMBOA-Glc accumulates in the insect hemolymph and can be reactivated upon attack by nematodes to form MBOA [230], a toxic compound for both EPNs and their endosymbiotic bacteria [230]. By comparing EPN populations from the primary (US and Mexico) and invasive (Europe, Asia, Africa) ranges of the herbivore and conducting real-time selection assays, Zhang et al. [231] demonstrated that the herbivore adaptation to hijack plant defenses can shape the evolution of resistance in nematodes. Although *H. bacteriophora* EPNs from the invasive WCR range were again repelled by MBOA-Glc and susceptible to HDMBOA-Glc, EPNs from the original range of WCR were neither repelled nor susceptible to the sequestered compounds. Rearing a susceptible EPN strain in benzoxazinoid-sequestering hosts was sufficient for the nematode to evolve a complete resistance to benzoxazinoid-dependent defenses. The ability of EPNs to overcome these defenses was further associated with higher infectivity rates of WCR. Similarly, a screening of *H. bacteriophora* Mexican isolates showed that most were resistant to the benzoxazinoid defenses of WCR larvae [232]. Interestingly, the variability in infectiveness of these isolates in the benzoxazinoid-sequestering WCR larvae suggests other WCR defensive mechanisms may exist and require further investigation. The relative contribution of genetic variation and epigenetic effects in the nematodes and its endosymbionts is currently under investigation. Early results demonstrate that engineering bacterial symbionts that are resistant to the WCR benzoxazinoid-defenses can improve EPN infectivity [233]. Resistance to benzoxazinoids of five *Photorhabdus* strains was successfully enhanced through experimental evolution. Strikingly, the evolution of resistance was acquired through multiple mechanisms in the different bacterial strains, because the observed insertions and nonsynonymous point mutations did not overlap. The insertions and mutations were located in genes encoding for a DNA-directed RNA polymerase, a transcriptional regulator of porins, a regulator of unsaturated fatty acid biosynthesis, a ligase involved in the biosynthesis of the outer membrane, and in an aquaporin gene, *aqpZ*, involved in membrane permeability. Further characterization of *aqpZ* confirmed its impact in benzoxazinoid resistance, as complementation of the mutated strain with the wild-type gene restored the bacterial susceptibility to MBOA. Reestablishing symbiosis between EPNs and the enhanced *Photorhabdus* strains increased EPN infectivity towards WCR by over 50%. Efforts are now underway to test this strategy in the field and to assess whether EPNs compatible with commercial application can be enhanced in this manner.

The growing body of literature documenting factors shaping EPN success in killing WCR larvae will surely enhance the efficacy of EPN-mediated strategies. Using attractant signals for EPNs may allow for the maintenance of elevated numbers of EPNs in the field. This solution was tested with transgenic maize plants that constitutively release *(E)*-β-caryophyllene, which resulted in effective WCR suppression in the presence of EPNs [234]. However, the overexpression of the associated terpene synthase also had a number of physiological and ecological costs [235]. Releasing the pure compound synthetically may be an alternative option, but the long-term impact of the sometimes-deceptive strategy requires careful investigation. Adding EPN-infected cadavers that attract both WCR larvae and EPNs [226,227] is a promising avenue, at least for high-value smaller fields. Placing EPN-infected cadavers in the field confers multiple advantages as the insect cadavers will provide the EPNs with shelter until favorable soil conditions are reached [236], thereby optimizing EPN survival, dispersal, and virulence after application [237,238]. In a greenhouse assay, Shapiro-Ilan et al. [238] demonstrated that the release of EPN-infected insect cadavers reduced the survival of the root weevil, *D. abbreviates*, and the black vine weevil, *Otiorhynchus sulcatus*, two times better than EPN suspensions within 7 days. The application of EPN-infected cadavers eliminated the herbivore population within 28 days, whereas EPN suspensions only reduced the herbivore populations by about 50%. Because EPN-infected cadavers induce plant resistance against leaf pathogens through volatile chemical cues [228,229], this strategy may be valuable for IPM. Alternatively, it is possible to apply nematodes in encapsulated hydrocapsules containing EPN quiescence factors covered with herbivore attractants and feeding stimulants. Such a strategy would attract WCR larvae to feed on the capsules, thereby directly releasing EPNs [239,240]. When applied in the field, these hydrocapsules effectively controlled WCR populations and reduced damage [240]. Deployment of EPN-containing hydrocapsules for control also appears feasible in other herbivore species [220]. Finally, priming, selecting, or engineering EPNs for increased responsiveness to WCR-indicating chemical cues or for increased resistance to WCR defenses appears promising. Priming of EPNs can be achieved through exposure to insect cues, such as insect macerates or pheromones, prior to application in the field [241,242,243]. Selection of EPNs or their endosymbiotic bacteria for enhanced responsiveness or resistance to insect chemicals can be obtained within a few generations in laboratory conditions [231,233]. The growing understanding of EPN biology and of their interactions with prey will only enhance the efficacy of integrated pest management strategies in general.

## 9. Conclusions

**Figure 1:**
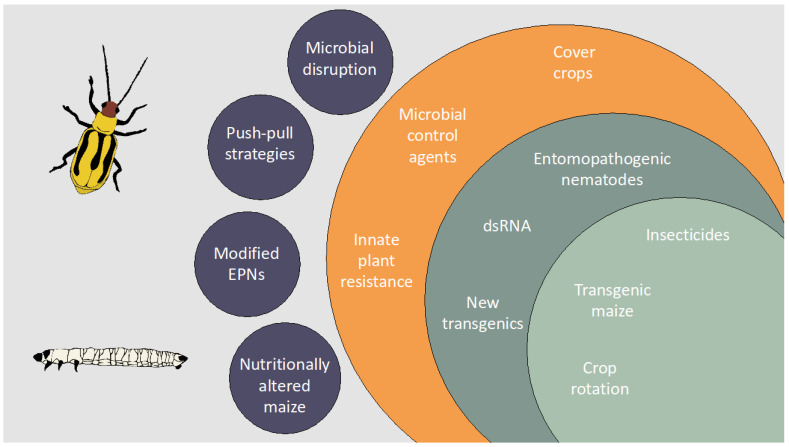
Broadening of management tactics aimed at controlling western corn rootworm (*Diabrotica virgifera virgifera* LeConte) and reducing damage to maize. The inner most circle (light green) represents the most common and widely adopted management techniques, all of which have seen failures in the field due to evolved resistance by western corn rootworm. The middle circle (dark green) represents management techniques less frequently adopted but have demonstrated effectiveness in laboratory or small field trials. The outermost circle (orange) represents management tactics that show promise and could be adopted using existing technology. Orbiting circles (blue) represent future management tactics that could be used if developed further.

Reliance on one dimensional management techniques has resulted in failures, both in terms of technologies and population suppression. In dealing with this established and persistent pest in the US and Europe, additional tools and a multifaceted approach to management are needed (Figure 1). The intense research involving WCR biology and chemical ecology has yielded knowledge that could translate into effective management strategies in the near- and long-term. Here, we reviewed a number of potential WCR management possibilities that, if implemented, have promise for new, effective, and sustainable WCR management. Results from lab-based studies sometimes fail to translate to field-based studies. Many of the strategies discussed here need additional testing in field settings under varying environmental conditions to properly assess their commercial viability but hold promise nonetheless.

## Figures and Tables

**Table 1:** Breeding efforts to develop native plant defense to the western corn rootworm over the past 85 years.

Years	Location	References
1935–1945	Illinois Natural History Survey	[61,62]
1970–2007	Iowa State University	[63,64,73,84,95,106,108,109,110,111,112]
1963–2010	USDA-ARS, Brookings, South Dakota	[65,66,67,68,69,70,71,72,74,75,76,77,78,79,80,81,113]
1990–1997	University of Ottawa	[82,83,85,86,87,88,89]
1992–present	USDA-ARS, Columbia, MO	[90,91,92,93,94,96,97,98,99,100]
1995–present	University of J. J. Strossmayer	[101,102,103,104,114]
2002–present	University of Illinois-Champaign	[105,107]

## Data Availability

Not applicable.
